# Bacteriophage treatment of disseminated cutaneous *Mycobacterium chelonae* infection

**DOI:** 10.1038/s41467-022-29689-4

**Published:** 2022-05-03

**Authors:** Jessica S. Little, Rebekah M. Dedrick, Krista G. Freeman, Madison Cristinziano, Bailey E. Smith, Constance A. Benson, Tulip A. Jhaveri, Lindsey R. Baden, Daniel A. Solomon, Graham F. Hatfull

**Affiliations:** 1 Division of Infectious Diseases, Brigham and Women’s Hospital, Boston, MA USA; 2 Harvard Medical School, Boston, MA USA; 3 Dana-Farber Cancer Institute, Boston, MA USA; 4 Department of Biological Sciences, University of Pittsburgh, Pittsburgh, PA USA; 5 Division of Infectious Diseases & Global Public Health, University of California San Diego, San Diego, CA USA; 6 Division of Medical Microbiology, Brigham and Women’s Hospital, Boston, MA USA; 7 Division of Infectious Diseases, University of Mississippi Medical Center, Jackson, MS USA

**Keywords:** Bacteriophages, Bacterial infection

## Abstract

*Mycobacterium chelonae* is a rare cause of chronic disseminated cutaneous infections in immunocompromised patients. Multidrug-resistant *M. chelonae* infections present a challenge for treatment, and prolonged antimicrobial courses lead to significant toxicities and further antimicrobial resistance. We report a case of refractory cutaneous disseminated *M. chelonae* infection in a patient with seronegative arthritis on immunotherapy with tofacitinib that was treated with combination antimicrobial, surgical, and single bacteriophage therapy with excellent clinical response. The patient developed neutralizing antibodies against the bacteriophage but continues to have stable improvement of disease with negative biopsies and no evidence of bacterial resistance to the phage.

## Abstract (web summary)

Increasing rates of multidrug-resistant bacterial infections has renewed interest in the therapeutic use of phages. Here the authors report an individual with cutaneous M. chelonae infection, and the improvement of disease upon treatment with a bacteriophage in combination with antimicrobial therapy.

## Introduction

*Mycobacterium chelonae*, a rapidly growing nontuberculous mycobacterium, is a rare cause of chronic infections in immunocompromised hosts^1,2^. This organism is ubiquitous in the environment and most frequently manifests clinically as localized skin or soft tissue infection or disseminated cutaneous disease^1,3,4^. Nosocomial infections can occur following procedures such as plastic surgery or exposure to contaminated equipment or substances, both in healthcare settings and community settings such as tattoo parlors^5–8^. Catheter-related bloodstream infections, septic arthritis, and osteomyelitis are also reported, while pulmonary disease is less common^1^. Disseminated cutaneous disease occurs most commonly in immunosuppressed patients including those who have undergone solid organ transplantation or with autoimmune disorders receiving treatment with corticosteroids or immunotherapy^1,9,10^. Similar to other mycobacterial species such as *Mycobacterium abscessus*, widely known for extensive antimicrobial resistance, *M. chelonae* has also been demonstrated to be multidrug-resistant^1,10–12^. Here we report the clinical course of a man with refractory disseminated cutaneous *M. chelonae* infection who was treated with a single bacteriophage (Muddy) in combination with antimicrobial therapy with excellent clinical response. To our knowledge this is the first case of *M. chelonae* infection treated with bacteriophage therapy.

## Results

### Clinical course

A 56-year-old man presented to dermatology clinic in January of 2020 with new nodular lesions on his left upper extremity as well as weight loss, night sweats, myalgias, and fatigue. He had a history of stage II chronic kidney disease, myxomatous mitral valve prolapse requiring mitral valve repair in 2014, and seronegative arthritis involving his bilateral hands, wrists, knees, and ankles diagnosed in 2019, as well as peripheral neuropathy of unclear etiology that was diagnosed simultaneously with his arthritis. Over the year preceding his presentation, he was treated with multiple immunologic therapies for his arthritis including methotrexate, etanercept, adalimumab, and most recently tofacitinib since November 2019 with minimal response. He also received multiple courses of prednisone up to 40 mg per day. At the time of presentation, he was on treatment with 10 mg per day of oral prednisone.

**Fig. 1:**
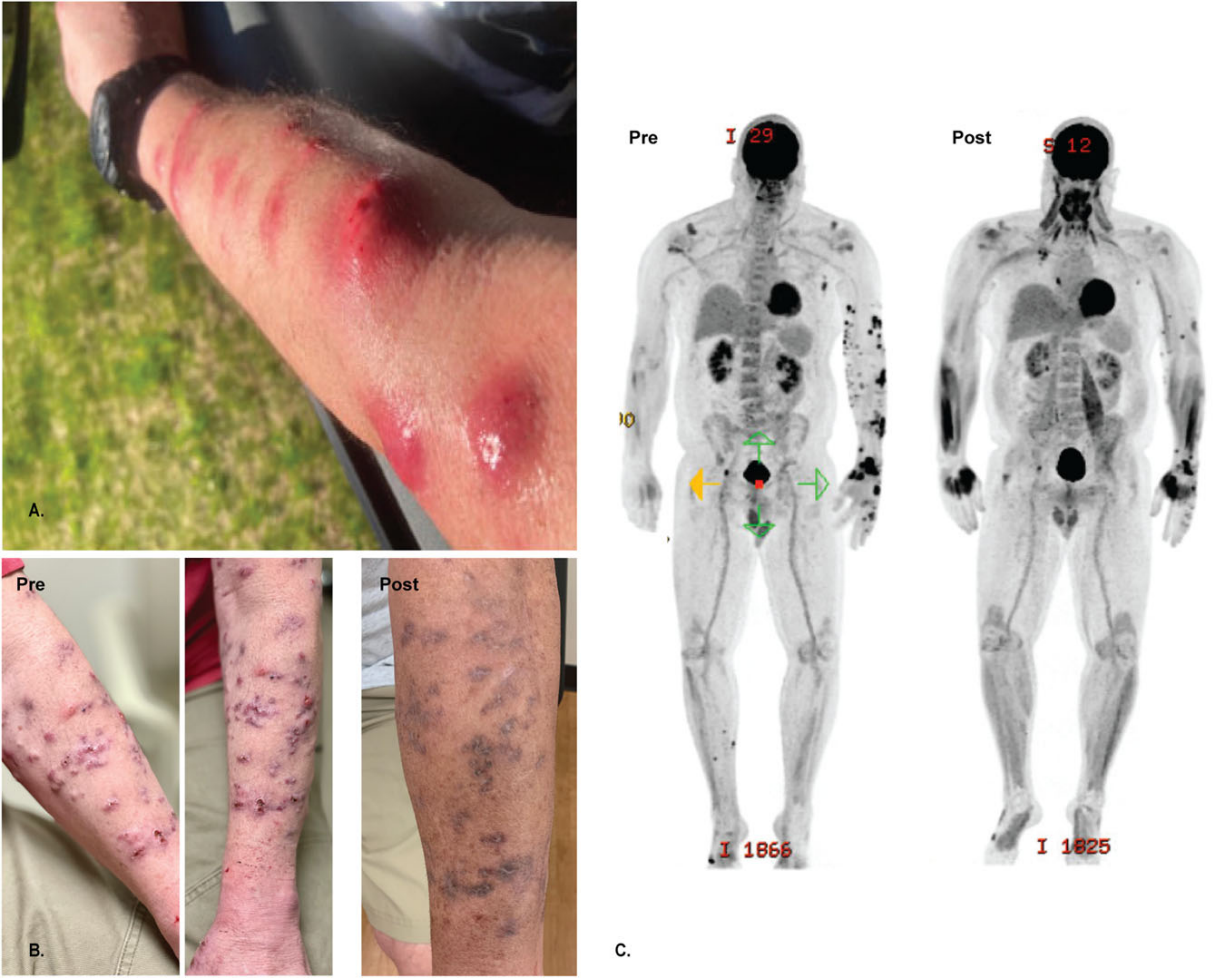
Clinical course of *M. chelonae* infection. **A** Left upper extremity with multiple large erythematous, fluctuant to nodular lesions ultimately diagnosed as disseminated cutaneous *Mycobacterium chelonae* infection. **B** Images of the left upper extremity lesions prior to (Dec 2020) and following (August 2021) addition of bacteriophage therapy. **C** PET/CT prior to (March 2021) and following (August 2021) addition of bacteriophage therapy.

**Fig. 2:**
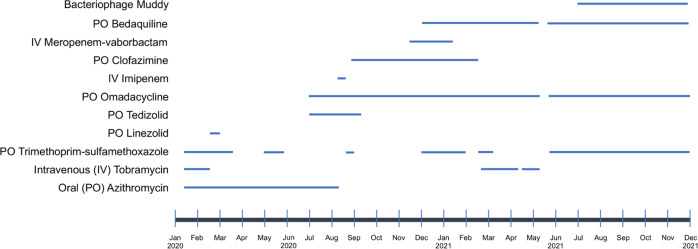
Treatment of *M. chelonae* infection. Timeline of antimicrobial and bacteriophage administration for *M. chelonae* infection from January 2020 to December 2021.

In the clinic, he described several months of waxing and waning nodular lesions on the left upper extremity with intermittent spontaneous drainage (Fig. 1A–B). He denied any known trauma or penetrating injury to that region. A biopsy was performed with mycobacterial cultures that grew *M. chelonae*. Pathology demonstrated abundant *Mycobacteria* on staining with dense infiltrates of admixed inflammatory cells and necrosis. The organism susceptibilities are presented in Table 1. The patient’s course of antimicrobial treatment has been characterized by a series of severe toxicities resulting in modification of treatment (Fig. 2). The patient started treatment with oral azithromycin, oral trimethoprim-sulfamethoxazole (TMP/SMX) and intravenous tobramycin on January 23, 2020. His course was complicated by hospitalization in late February with worsening symptoms thought to be due to immune reconstitution inflammatory syndrome in the setting of tapering prednisone. Further immunologic therapy including tofacitinib was held. He also developed multiple medication toxicities including renal injury and ototoxicity due to tobramycin and gastrointestinal symptoms due to TMP/SMX, leading to a period of azithromycin monotherapy and the development of subsequent resistance. Mycobacterial isolator cultures were negative, but in June 2020 he developed new lesions in the periumbilical subcutaneous tissue as well as on the right lower extremity indicative of disseminated cutaneous infection. A positron emission tomography/computed tomography (PET/CT) scan showed no visceral sites of disease (Fig. 1C), and a transthoracic echocardiogram was negative for valvular vegetations. Workup was also notable for the presence of hypogammaglobulinemia and a variant of uncertain significance in the NLRP12 gene on whole exome sequencing, which has been associated with immune dysregulation and familial autoinflammatory disorders. He had no other evidence of underlying immunodeficiencies. He was treated with several new antimicrobial medications over the subsequent months including tedizolid, omadacycline, clofazimine, bedaquiline, imipenem, TMP/SMX, and meropenem-vaborbactam, that were limited by toxicities or emergence of resistance as shown in Table 1 and Fig. 2. He had persistent skin lesions with positive mycobacterial cultures and positive mycobacterial staining on pathology in May, August, and October of 2020 despite efforts to optimize his antimicrobial regimens. In December 2020, he developed severe left wrist pain and was found to have septic arthritis of the left wrist. He underwent a left wrist synovectomy with irrigation and debridement on December 14, 2020 as well as debulking of his most active skin lesions with pathology that demonstrated necrotizing granulomas, though cultures remained negative. He underwent reinduction with a 5-drug regimen of meropenem-vaborbactam, TMP/SMX, omadacycline, clofazimine, and bedaquiline for one month and was then transitioned to a 3-drug regimen of omadacycline, clofazimine, and bedaquiline. He continued on this regimen until February 2021, when he developed worsening nodular and fluctuant lesions, and repeat biopsy demonstrated florid dermal abscesses with numerous *Mycobacteria* on Fite and acid-fast bacilli (AFB) stains. Given his refractory infection, the patient was identified as a potential candidate for bacteriophage therapy.

**Table 1:** Evolution of *Mycobacterium chelonae* susceptibilities.

*M. chelonae* Susceptibilities:January 2020		*M. chelonae* Susceptibilities:October 2020	
Agent	MIC^1^	Agent	MIC
Trimethoprim-sulfamethoxazole	0.5/9.5	Trimethoprim-sulfamethoxazole	1/19
Linezolid	≤1	Linezolid	4
Ciprofloxacin	2	Ciprofloxacin	>4
Imipenem	16	Imipenem	>64
Moxifloxacin	2	Moxifloxacin	4
Cefoxitin	>128	Cefoxitin	>128
Amikacin	16	Amikacin	16
Doxycycline	>16	Doxycycline	>16
Minocycline	>6	Minocycline	>8
Tigecycline	0.03	Tigecycline	0.12
Tobramycin	≤1	Tobramycin	—
Clarithromycin	NR	Clarithromycin	>16
Clofazimine	—	Clofazimine	<0.015
Bedaquiline	—	Bedaquiline	0.001
Omadacycline	—	Omadacycline	0.06

^1^ Minimum inhibitory concentration

### Identification of suitable therapeutic phages

**Fig. 3:**
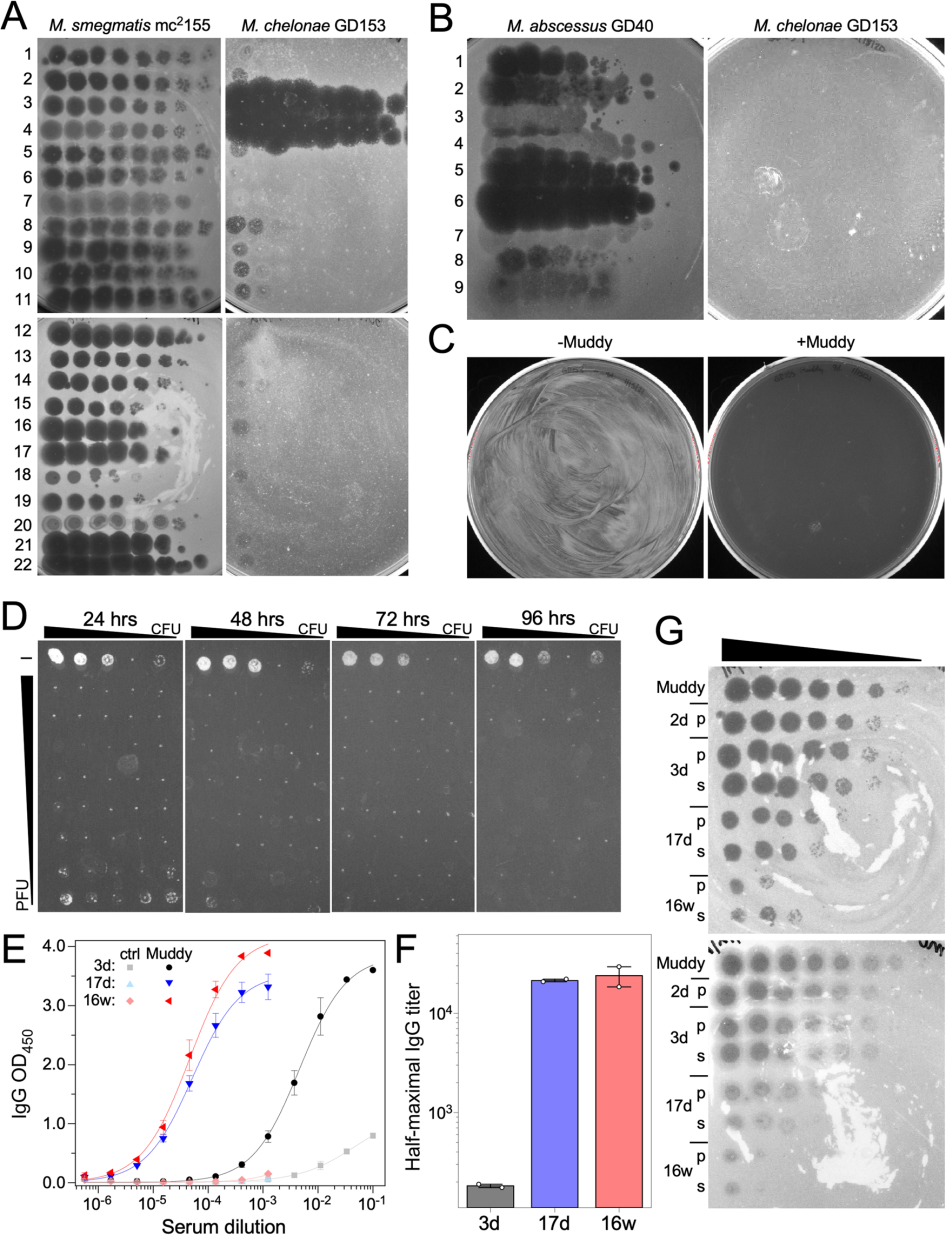
Bacteriophages for therapy of *M. chelonae*. **A** Ten-fold serial dilutions of each phage (left) were spotted onto top agar overlays of *M. smegmatis* mc^2^155 and *M. chelonae* GD153. Phages: 1, BPs∆*33*HTH_HRM10; 2, BPs∆*33*HTH_HRM^GD03^; 3, Muddy; 4, Muddy_HRM^GD04^; 5, Itos; 6, Adephagia∆*41*∆*43*; 7, ZoeJ∆*45*; 8, Fionnbharth∆*45*∆*47*; *9*, Elmo_HRM^mc2155^; 10, Isca_cpm; 11, Fred313cpm_∆*33*; 12, CrimD∆*41*-*43*; 13- BPs∆*33*HTH_HRM10_REM1; 14, Faith1∆*38-40*; 15, Faith1∆*38-40*_HRM^GD69^; 16, MissWhite; 17, MissWhite-D29_Hybrid1, 18, Maco6; 19, TM4; 20, Wildcat; 21, D29; and 22, D29_HRM^GD40^. **B** As for panel A but using *M. abscessus* lytically induced prophages plated on *M.* *chelonae* GD153 and control strain *M. abscessus* GD40. Phages: 1, phiGD20-1; 2, phiGD22-1; 3, phiGD23-1; 4, phiGD21-1; 5, phiGD34-2; 6, phiGD89-1; 7, phiGD57-1; 8, phiGD17-1; 9, phiGD24-3. **C** Killing of *M. chelonae* GD153 by Muddy following infection in liquid culture (moi, 10); -Muddy, no phage control. **D** Efficient killing of *M. chelonae* GD153 over ranges of bacterial and phage concentrations. All rows contain 10-fold serial dilutions of bacterial culture. Top row (–), no phage control; rows 2-9, 10-fold serial diltions, with 10^10^ PFU in row 2. **E** ELISA responses for anti-Muddy IgG antibodies in sera from 3 days, 17 days or 16 weeks after phage treatment initiation. Negative controls (ctrl) for uncoated wells are also shown. *n* = 2 technical replicates are shown. Data points are the average of two independent sets of serum dilutions and measurements, with error bars ± one standard deviation. **F** Half-maximal IgG titers derived from ELISA curve fits in panel D. Duplicate measurements are shown as points; bar height is the mean titer with error bars±one standard deviation. Source data for panels E and F are provided as a Source Data file. **G** To test for antibody neutralization, Muddy was incubated with either plasma (p) or serum (s) from 2 days, 3 days, 17 days or 16 weeks after the start of phage administration for 2 h (top) or 24 h (bottom) and then 10-fold serial dilutions were spotted onto top agar overlays of *M. smegmatis* mc^2^155. Plates were incubated at 37 °C for 48 h.

The *M. chelonae* isolate from October 2020 (designated strain GD153) was used to screen for potentially therapeutic phages. GD153 was tested for sensitivity to a panel of ~20 *Mycobacterium smegmatis* phages that have previously shown potential for activity against *M. abscessus* infections (Fig. 3A), as well as nine lytically growing phages induced from lysogenic *M. abscessus* clinical isolates (Fig. 3B)^13^. *M. chelonae* GD153 does not grow at 37 °C and all phage assays were performed at 30 °C. A single phage—Muddy (and its host range variant Muddy_HRM^GD04^) – efficiently infects GD153 (Fig. 3A). Phage Muddy is lytic and efficiently kills *M. chelonae* GD153 with no evident survival after challenging 4 ×10^8^ cells with phage at a multiplicity of infection of ten (Fig. 3C). Muddy also kills *M. chelonae* GD153 well over a broad range of bacteria and phage concentrations (Fig. 3D). Muddy has a siphoviral morphology, grows well to high titer, is stable (maintains titer), efficiently kills *M. tuberculosis*, and has been used therapeutically to treat *M. abscessus* infections^14,15^. Muddy was grown to high titer on *M. smegmatis*, highly purified, and shown to be endotoxin-free and sterile, as described previously^14,15^. Regulatory and institutional approval for intravenous mycobacteriophage administration was obtained from the Massachusetts General Hospital/Brigham and Women’s Hospital institutional review board and the U. S. Food and Drug Administration via a single patient expanded access application.

### Phage administration and patient response

In the month preceding initiation of bacteriophage therapy, the patient continued on treatment with omadacycline, bedaquiline, and TMP/SMX. He underwent a second debulking surgery of his largest skin lesions on June 10, 2021 with pathology that demonstrated abundant necrotizing granulomas that stained positive for AFB. He started intravenous bacteriophage therapy twice daily at a dose of 10^9^ plaque-forming units (PFUs) per dose of Muddy on June 15, 2021 in conjunction with his prior antimycobacterial therapy. He reported subjective flushing after each dose that lasted up to an hour, but denied fevers, chills, rigors, respiratory symptoms, gastrointestinal symptoms, or neurologic symptoms. He remained hemodynamically stable with stable laboratory markers. Two days after initiation, he experienced chills and nausea two hours after infusion of the bacteriophage without other associated symptoms or vital sign changes. These findings resolved without intervention. He was monitored with examination and weekly laboratory testing including a basic metabolic panel, liver function tests, and complete blood count with differential following discharge from the hospital on June 18, 2021, where he was noted to be doing well without any reported adverse effects and stable laboratory markers. His skin lesions improved significantly over the first two weeks of therapy and continued to show steady improvement over the subsequent months with decreased inflammation and nodularity (Fig. 1B). Small subcutaneous nodules persisted, so repeat biopsies were performed in August and November 2021, and showed no evidence of granulomas or AFB on histopathology and tissue cultures were negative. A repeat PET/CT in September 2021 demonstrated significant interval improvement of size and intensity of FDG uptake in previously seen cutaneous and subcutaneous soft tissue lesions (Fig. 1C).

Serum from days 3 and 17, and week 16 after initiation of phage administration was tested for the presence of antibodies against Muddy (Fig. 3E, F). The day 3 sample showed a weak but specific IgG response that presumably pre-dated phage administration, but at both day 17 and week 16 post-phage initiation there was a robust IgG-mediated response, comparable to that reported in a prior patient^15^. The antibody response at 17-days was mildly neutralizing but more potently neutralizing at 16 weeks (Fig. 3G). These antibody responses did not negate the overall clinical improvement of the patient. Given his marked improvement and lack of adverse effects, the patient has continued on intravenous bacteriophage therapy. He has had intermittent flares of his seronegative arthritis and additional immunosuppressive therapy is planned with concern that alteration in the host immune system could impact control of infection, bacteriophage and antibiotics have been continued. He will continue on combination therapy for treatment of *M. chelonae* until at least 4 – 6 weeks after initiation of additional immunosuppressive treatments with the plan to sequentially discontinue the bacteriophage followed by antibiotics thereafter. The patient consented to the publication of this information.

## Discussion

Bacteriophages are organisms that are ubiquitous in the environment with the ability to infect and kill bacterial hosts^16–18^. Bacteriophage therapy using a cocktail of multiple phage isolates has been recently successfully used for the treatment of resistant bacterial infections including multidrug-resistant nontuberculous mycobacterial infections in conjunction with traditional antibiotic therapy^14,15,19,20^. Widespread use of bacteriophage therapy is limited by logistic and regulatory challenges as well as the development of bacterial resistance or neutralizing antibodies that may reduce host response to the bacteriophage^15,21,22^.

Here, we describe the novel treatment of a patient with disseminated cutaneous *Mycobacterium chelonae* infection with a single bacteriophage in conjunction with antibiotic and surgical management. While he had previously received extensive courses of antimicrobials as well as prior surgical debridement in December 2020, his skin lesions and biopsies remained culture positive with evidence of necrotizing granulomas and abundant mycobacteria on staining. Following initiation of bacteriophage therapy, the skin lesions significantly improved both on examination and radiographically on PET/CT scan. Furthermore, two biopsies at 2 and 5 months post-treatment have demonstrated no evidence of granulomas or AFB on histopathology and tissue cultures have remained negative. The patient has had no adverse events related to his bacteriophage therapy and has successfully administered this therapy intravenously at home for > 6 months.

A potential major limitation of bacteriophage therapy is the development of phage resistance, which can potentially be countered using an appropriately-designed phage cocktail^14,19^. Here, we were limited to a single phage as no other phages tested were highly active against the patient’s strain of *M. chelonae*. Although resistance to Muddy is likely to occur, it was not detected in vitro, consistent with the infrequency of phage resistance in *M. abscessus* isolates^13^. Moreover, phage resistance in vivo leading to loss of treatment efficacy was not observed. These observations suggest that phage resistance of NTM pathogens may not be the impediment encountered with other pathogens.

A second barrier to the successful treatment of bacterial infections with phage therapy is the complex interaction between the host immune system and the bacteriophage^15,22^. The development of neutralizing antibodies has previously been reported, though the clinical consequences remain unclear^23^. In one case by Dedrick at al., the emergence of neutralizing antibodies correlated temporally with treatment failure, however other reports have shown favorable outcomes resulting from bacteriophage therapy despite the development of anti-phage antibodies^15,22^. In our case, the patient has maintained stably improved disease and negative microbiologic and histopathologic studies despite the identification of a neutralizing antibody response to the phage. It is plausible that the bacteriophage acted to rapidly decrease the burden of infection, leading to improved control with ongoing antimicrobial therapy that had previously been insufficient. Another consideration is that phage replication became self-sustaining at the sites of infection, such that administration subsequent to the onset of the neutralizing response had little effect and was unnecessary.

To our knowledge, this is the first case of a human *M. chelonae* infection treated with a bacteriophage, and the first case of bacteriophage treatment with a single phage for a mycobacterial infection. Bacteriophage therapy is a promising therapeutic option for multi-drug resistant infections, though improved understanding of safety, the factors driving the development of bacterial resistance, and the clinical significance of antibody-mediated phage neutralization is vital to advance this therapeutic option for patients.

## Methods

### Bacterial strains

*M. smegmatis* mc^2^155 was grown as previously described.^24^ *M. chelonae* GD153 and *M. abscessus* GD40 were grown in Middlebrook 7H9 media with OADC and 1 mM CaCl_2_, shaking, at 30 °C for 10-12 days and 37 °C for 4-5 days, respectively^13^. For plaque assays, *M. chelonae* and *M. abscessus* cultures were sonicated briefly in a cup-horn sonicator (Q-sonica 500) at 30% amplitude with 15 s on and 10 s off until visibly dispersed ^14^.

### Phage susceptibility screening

A standard plaque assay was used to determine phage susceptibility of GD153, spotting 10-fold serial dilutions of each phage onto top agar overlays containing 500 µl of saturated cultures of each bacterial isolate.^24^ The most concentrated phage spot was typically 10^8^ -10^9^ PFU/ml; TM4 was 10^6^ PFU/ml. Phages used were primarily those shown previously to infect one or more strain of *M. abscessus* or which were recovered as induced prophages from *M. abscessus* clinical isolates^13,15^. Efficiencies of plaquing (EOP) were determined by comparing phage titers on *M. smegmatis* mc^2^155, *M. chelonae* GD153 and *M. abscessus* GD40 isolates, as described previously.^24^

### Therapeutic mycobacteriophage preparation

Phage Muddy was grown on *M. smegmatis* mc^2^155 using solid media using a top layer containing 0.35% agar. After phage growth, the whole top layer was collected and centrifuged to remove debris and cells. The clarified lysate (3 × 10^11^ PFU ml^−1^) was filtered through a 0.22 μm filter and the phage collected by centrifugation at 100,000 x g for one hour. The phage pellet was resuspended in phage buffer (68 mM NaCl, 10 mM Tris HCl pH 7.5, 10 mM MgSO_4_, 10 mM CaCl_2_) and cesium chloride (CsCl) was added to a density of 1.5 g cm^−3^ (4.1 M), and subjected to equilibrium density gradient centrifugation at 132,600 x g for 16 h. The visible phage band was collected (1-3 ml), the volume increased with CsCl (1.5 g cm^−3^) and similarly centrifuged. The visible phage band was collected and stored at 4 °C; this yielded ~2 ml of purified phage with a titer of ~10^13^ PFU ml^−1^. One ml of Muddy was dialyzed against 1 L of Ringers Solution (Oxoid) four times for a minimum of 4 hours each, and endotoxin shown to be below the level of detection using the EndoZyme II (Hyglos GmbH) assay. USP71 sterility testing was completed by Accugen. Vials containing 0.1 ml of Muddy at 1 ×10^11^ PFU/ml were resuspended in 10 ml of Ringers Solution. One ml of this solution was used for each dose (1 ×10^9^ PFU). Each batch of phage prepared was titered to check for correct concentration, subjected to an Endotoxin assay to look for any contaminating lipopolysaccharide, and sent to Accugen Laboratories for sterility testing.

### Phage neutralization assays

Plasma or serum samples were incubated with phage by adding 10 µl of plasma or serum to 90 µl of phage buffer containing ~1×10^10^ PFU of phage Muddy. After incubating at room temperature for 2 or 24 hours, 10-fold serial dilutions were prepared and 3 µl of each dilution spotted onto top agar overlays containing *M. smegmatis* mc^2^155. Plates were incubated at 37 °C for 24 hours.

### Elisa

Enzyme-linked immunosorbant assays (ELISAs) were performed as described previously^14^. In brief, EIA microplates (Corning CLS3590) were coated overnight with 100 µl of coating buffer (carbonate-bicarbonate with pH = 9.6, Sigma C3041) or phage Muddy diluted to 5 ×10^9^ pfu ml^−1^ in coating buffer. After incubation, washing, and blocking, 100 µl of diluted patient serum was added and incubated at 4 °C for ~20 hours. Anti-Muddy IgG was probed by incubation with goat Anti-Human IgG Fc (HRP) pre-adsorbed (Abcam ab98624) diluted 1:10,000, and detected following addition of 100 µl of 3,3′,5,5′-Tetramethylbenzidine (TMB) substrate (Sigma T0440), an 8 min incubation, and addition of 2 N H_2_SO_4_ to stop the reaction. Absorbance at 570 nm (background) was subtracted from absorbance at 450 nm (signal) and the difference was plotted against the serum dilution and fit with a fixed-baseline logistic curve; half-maximum IgG titers were determined from the curves.

### Statistics and reproducibility

The Origin 2021b (64-bit) SR2 9.8.5.212 software was used for ELISA data analysis. Fixed-baseline logistic functions were used to fit the data, as shown in Fig. 3E. Half-maximal serum dilution is a parameter of these fits, and was inverted to yield half-maximal titer (Fig. 3F). ELISA curves in Fig. 3E are averages of two technical replicates, with error bars showing one standard deviation from the mean. Half-maximal titers for the two replicates are shown as white datapoints in Fig. 3F, with bars indicating the average of two replicates and error bars of one standard deviation.

The screen for infection by *M. smegmatis* phages (Fig. 3A) was performed three times and Fig. 3A shows representative data. The screen using lytically propagated prophages (Fig. 3B) and the killing/survival assays (Fig. 3C, D) were performed once. Neutralization assays were performed twice and representative data are shown in Fig. 3G.

### Clinical procedures

This research protocol was approved by the Massachusetts General Hospital/Brigham and Women’s Hospital institutional review board and the U.S. Food and Drug Administration via a single patient expanded access application. The patient provided informed consent, according to CARE guidelines and in compliance with the Declaration of Helsinki principles

### Reporting summary

Further information on research design is available in the Nature Research Reporting Summary linked to this article.

## Supplementary information

Reporting Summary

## Data Availability

The datasets generated during and/or analyzed during the current study are available from the corresponding author on reasonable request. Source data are provided with this paper.

## Source data

Source Data
