# Supplementary material for: Bacteriophage treatment of disseminated cutaneous Mycobacterium chelonae infection
Source: Nat Commun. 2022 May 3;13:2313. doi: 10.1038/s41467-022-29689-4 (PMC9064978; doi:10.1038/s41467-022-29689-4)
Supplement: Supplementary file 1 — Reporting Summary [file 41467_2022_29689_MOESM1_ESM.pdf]

nature portfolio

Corresponding author(s): Jessica S. Little

Last updated by author(s): Mar 4, 2022

# Reporting Summary

Nature Portfolio wishes to improve the reproducibility of the work that we publish. This form provides structure for consistency and transparency in reporting. For further information on Nature Portfolio policies, see our Editorial Policies and the Editorial Policy Checklist.

## Statistics

For all statistical analyses, confirm that the following items are present in the figure legend, table legend, main text, or Methods section.

| n/a | Confirmed | |
|---|---|---|
| ☐ | ☒ | The exact sample size ($n$) for each experimental group/condition, given as a discrete number and unit of measurement |
| ☐ | ☒ | A statement on whether measurements were taken from distinct samples or whether the same sample was measured repeatedly |
| ☒ | ☐ | The statistical test(s) used AND whether they are one- or two-sided<br>*Only common tests should be described solely by name; describe more complex techniques in the Methods section.* |
| ☒ | ☐ | A description of all covariates tested |
| ☒ | ☐ | A description of any assumptions or corrections, such as tests of normality and adjustment for multiple comparisons |
| ☐ | ☒ | A full description of the statistical parameters including central tendency (e.g. means) or other basic estimates (e.g. regression coefficient) AND variation (e.g. standard deviation) or associated estimates of uncertainty (e.g. confidence intervals) |
| ☒ | ☐ | For null hypothesis testing, the test statistic (e.g. $F$, $t$, $r$) with confidence intervals, effect sizes, degrees of freedom and $P$ value noted<br>*Give P values as exact values whenever suitable.* |
| ☒ | ☐ | For Bayesian analysis, information on the choice of priors and Markov chain Monte Carlo settings |
| ☒ | ☐ | For hierarchical and complex designs, identification of the appropriate level for tests and full reporting of outcomes |
| ☒ | ☐ | Estimates of effect sizes (e.g. Cohen's $d$, Pearson's $r$), indicating how they were calculated |

*Our web collection on statistics for biologists contains articles on many of the points above.*

## Software and code

Policy information about availability of computer code

| | |
|---|---|
| Data collection | No software used for data collection |
| Data analysis | Origin 2021b (64-bit) SR2 9.8.5.212 |

For manuscripts utilizing custom algorithms or software that are central to the research but not yet described in published literature, software must be made available to editors and reviewers. We strongly encourage code deposition in a community repository (e.g. GitHub). See the Nature Portfolio guidelines for submitting code & software for further information.

## Data

Policy information about availability of data

All manuscripts must include a data availability statement. This statement should provide the following information, where applicable:

- Accession codes, unique identifiers, or web links for publicly available datasets
- A description of any restrictions on data availability
- For clinical datasets or third party data, please ensure that the statement adheres to our policy

Source data are provided with this paper. Additional data generated during and/or analyzed during the current study are available from the corresponding author on reasonable request.

# Field-specific reporting

Please select the one below that is the best fit for your research. If you are not sure, read the appropriate sections before making your selection.

☒ Life sciences ☐ Behavioural & social sciences ☐ Ecological, evolutionary & environmental sciences

For a reference copy of the document with all sections, see nature.com/documents/nr-reporting-summary-flat.pdf

# Life sciences study design

All studies must disclose on these points even when the disclosure is negative.

| | |
|---|---|
| Sample size | This paper describes the outcome of one patient. The total number of phages tested was ~50. These ~50 phages have been sequenced and curated in the Hatfull lab as potentially useful for phage therapy, and are routinely used for screening infectivity of mycobacterial clinical isolates. |
| Data exclusions | No data were excluded from the analyses. |
| Replication | The patient treatment outcomes are not subject to replication. Technical replicates (# indicated in parenthesis) were performed for the IgG ELISA curves and endpoint titers (2). All replicates produced similar results, and displayed values are the mean +/- s.d. of replicates, where applicable. |
| Randomization | This is not relevant to our study because it describes the outcome of one patient. |
| Blinding | Blinding was not relevant to our study because only a single patient was treated |

# Reporting for specific materials, systems and methods

We require information from authors about some types of materials, experimental systems and methods used in many studies. Here, indicate whether each material, system or method listed is relevant to your study. If you are not sure if a list item applies to your research, read the appropriate section before selecting a response.

## Materials & experimental systems

| n/a | Involved in the study |
|---|---|
| ☐ | ☒ Antibodies |
| ☒ | ☐ Eukaryotic cell lines |
| ☒ | ☐ Palaeontology and archaeology |
| ☒ | ☐ Animals and other organisms |
| ☐ | ☒ Human research participants |
| ☒ | ☐ Clinical data |
| ☒ | ☐ Dual use research of concern |

## Methods

| n/a | Involved in the study |
|---|---|
| ☒ | ☐ ChIP-seq |
| ☒ | ☐ Flow cytometry |
| ☒ | ☐ MRI-based neuroimaging |

## Antibodies

| | |
|---|---|
| Antibodies used | Primary antibody source was patient serum. Secondary antibody source was Goat Anti-Human IgG Fc (HRP) pre-adsorbed (Abcam ab98624). |
| Validation | The manufacturer (Abcam) validates ab98624 for use in ELISAs (https://www.abcam.com/goat-human-igg-fc-hrp-preadsorbed-ab98624.html). |

## Human research participants

Policy information about studies involving human research participants

| | |
|---|---|
| Population characteristics | This is a 56 year-old man with cutaneous disseminated Mycobacterium chelonae infection who was treated under an emergency (compassionate use) IND |
| Recruitment | This is a 56 year-old man with cutaneous disseminated Mycobacterium chelonae infection who was treated under an emergency (compassionate use) IND |
| Ethics oversight | FDA single patient IND; Mass General Brigham Institutional Review Board |

Note that full information on the approval of the study protocol must also be provided in the manuscript.
